# Comparative study on the effect of hyperthermic massage and mechanical squeezing in the patients with mild and severe meibomian gland dysfunction: An interventional case series

**DOI:** 10.1371/journal.pone.0247365

**Published:** 2021-03-08

**Authors:** Dongkyun Han, Hoon Kim, Sehwan Kim, Yuli Park, Kyong Jin Cho

**Affiliations:** 1 Department of Ophthalmology, College of Medicine, Dankook University, Cheonan, Republic of Korea; 2 Beckman Laser Institute Korea, Dankook University, Cheoan, Republic of Korea; 3 Department of Biomedical Engineering, School of Medicine, Dankook University, Cheoan, Republic of Korea; Xiamen University, CHINA

## Abstract

**Background:**

Meibomian glands exist beneath the palpebral conjunctiva; thus, it is invisible to the naked eye without infrared imaging. This study used meibography to group patients with meibomian gland dysfunction (MGD) and assessed the effects of hyperthermic massage and mechanical squeezing in both groups.

**Materials and methods:**

Patients with MGD were divided into two groups, according to the degree of meibomian gland loss: group 1, in which the sum of eyelid scores ranged from 0 to 4 (mild to moderate gland loss) and group 2, in which the sum of eyelid scores ranged from 5 to 6 (severe gland loss). Hyperthermic massage and mechanical squeezing were given to both groups once a week for 4 weeks, and only non-preservative artificial tears were allowed. Ocular surface disease index (OSDI), Schirmer’s test, meibography score, tear break-up time (TBUT), ocular surface staining, expressible meibomian gland, and quality before and after treatment were compared.

**Results:**

Of the 49 patients who completed the 4 weeks of treatment and the evaluation at week 5, 29 were assigned to group 1 and 20 were assigned to group 2. Meibography scores, OSDI, TBUT, and expressibility of meibum had significant differences before and after treatments in both groups. However, there was no significant difference between the changes in clinical signs between group 1 and 2 after treatment. Without grouping, all patients showed significant decreases in meibography score, OSDI, cornea staining score, and increases in TBUT and expressibility of meibum after treatment.

**Conclusions:**

Considering the results of the current study, hyperthermic massage and mechanical squeezing may be effective in patients with meibomian gland loss, regardless of the degree of severity.

## Introduction

Meibomian gland dysfunction (MGD) is a chronic, diffuse abnormality of the meibomian glands and is commonly characterized by terminal duct obstruction and changes in glandular secretion [1–3]. With MGD, as a major cause of dry eye disease, patients may claim various symptoms depending on the eyelid inflammation and the degree of dry eye. MGD is usually caused by cornification of ductal epithelium and the increased viscosity of meibum [4–12]. Because meibomian glands exist under ocular conjunctiva and are only visible under infrared light imaging, it is necessary to perform meibography to accurately diagnose MGD and assess the meibomian gland condition.

Although ocular conditions are considered as well, mostly questionnaire-based indexes such as ocular surface disease index (OSDI), etc. are being used to categorize MGD patients in clinic for the assessment of severity of their symptoms [13]. However, such indexes may be altered when the patients have low sensitivity to pain and discomfort [14].

MGD patients receive appropriate treatments according to their grouping after categorized, and the treatments include physical treatments such as control over eyelid hygiene through patient education, lid scrub, and warm compresses, and chemical treatments such as the use of artificial tear drops, topical steroids, oral antibiotics, etc., depending on the patient’s symptoms [15]. Previous studies reported that thermodynamic treatment with LipiFlow^®^ for 3 months showed an improvement in MGD [13], and the symptoms in 96% of patients were relieved after 4 weeks of meibomian gland probing [16]. However, such physical treatments have been recommended mostly to the patients with mild to moderate MGD so far, and not considered for those with severe symptoms of MGD.

In this study, we categorized MGD patients into two groups according to their severity using meibography and assessed the effects of hyperthermic massage and expression treatment in both groups.

## Methods

The current study was conducted with the informed consent of subjects. This prospective, uncontrolled, open label, intervention study, followed the principles of the Declaration of Helsinki. The study protocol and informed consent were reviewed and approved by the Institutional Review Board of Dankook University Hospital before study initiation (IRB#DKUH201705003) and registered to Clinical Research Information Service (CRIS; http://cris.nih.go.kr) (KCT0005167).

All included MGD participants were diagnosed by evaluating conjunctival inflammation; clinical symptoms (ocular discomfort, itching or photophobia); fluorescein corneal and conjunctival staining; and clinical signs including lid margin abnormality, expressibility, and secretion quality; the diagnoses took place at Dankook University Hospital between June 2017 and December 2018. Exclusion criteria included a history of previous ocular or intraocular surgery, glaucoma or ocular hypertension, ocular infection, non-dry eye ocular inflammation, ocular allergy, autoimmune disease, wearing contact lenses during the study period, and the presence of current punctal occlusion. Pregnant or lactating women were also excluded. Individual characteristics of the included patients are in S1 Table.

Changes in the meibomian gland were observed with a Meiboviewer (Visual Optics, Chooncheon, Korea), and scored from 0 to 3 (Grade 0: no dropout of the meibomian gland; Grade 1: dropout of the meibomian gland below 1/3; Grade 2: dropout of the meibomian gland from 1/3 to 2/3; and Grade 3: dropout of the meibomian gland by >2/3). The study participants were divided into two groups according to the sum of upper and lower eyelid scores, as follows: group 1 with a total score of 0 to 4 (showing mild to moderate dropout of the meibomian gland) and group 2 with a total score of 5 to 6 (showing severe dropout of the meibomian gland). In each patient, the higher meibography score between both eyes was used for analysis.

Both groups were treated once a week with hyperthermic massage and mechanical squeezing with a cotton swab for a total of 4 weeks. Hyperthermic treatment was performed with an eyelid massage machine (Nurieye-5800, Seodong Medical, Busan, Korea) for 5 min, and mechanical squeezing of the upper and lower eyelid was performed by one physician with a cotton swab after local anesthesia with 5 mg/mL of proparacaine HCL (Alcaine, Alcon, Texas, USA). All patients used preservative-free sodium hyaluronate tear drops four times a day; no other eye drops were used during the study period.

Ocular surface disease index (OSDI), tear break-up time (TBUT), Schirmer’s test, corneal fluorescein staining, the expressibility of meibum, and quality of meibum were compared and analyzed at the fifth week of the study. All participants completed the validated 12-item OSDI questionnaire, which is assessed on a scale from 0 to 100, with higher scores representing greater disability. The total OSDI score was calculated using the following formula: OSDI score equals (sum of scores for all questions answered × 100) / (total number of questions answered × four) [17]. TBUT was measured per second from the initial blink under a cobalt-blue slit lamp with a fluorescein strip (Haag-Streit, Koeniz, Switzerland) to the time when a black dot or line, or the loss of fluorescein, was observed in the fluorescein-dyed tear layer. Measurements were repeated three times, and the average value was used for analysis. The expressibility of meibum was measured by counting the number of holes secreting lipid under squeezing of both upper and lower eyelids. Corneal fluorescein staining was categorized into six grades according to the Oxford grading scheme, based on the degree of corneal staining under yellow-filtered cobalt-blue light after fluorescein staining of the cornea [18]. The quality of meibum was evaluated according to the characteristics of the gland secretion and classified as one of four groups (1, clear; 2, cloudy; 3, toothpaste; and 4, no meibum). The average grade of both eyes was used for analysis.

Statistical analyses were conducted with SPSS 24 software (IBM, USA). The analyses included the chi-squared test for categorical variable analysis, Pearson’s correlation analysis among the variables, Levene’s test for equality of variance and Shapiro-Wilk normality test, independent samples t-test and Mann-Whitney U test, paired t-test and Wilcoxon’s signed rank test for changes in continuous variables before and after treatments. P<0.05 was considered significant. Graphs were created using the function *ggplot* and *ggline* from R version 4.0.3 (The R Foundation, Austria). Reporting in accordance with the TREND guidelines for non-randomized interventional trials.

## Results

**Fig 1:**
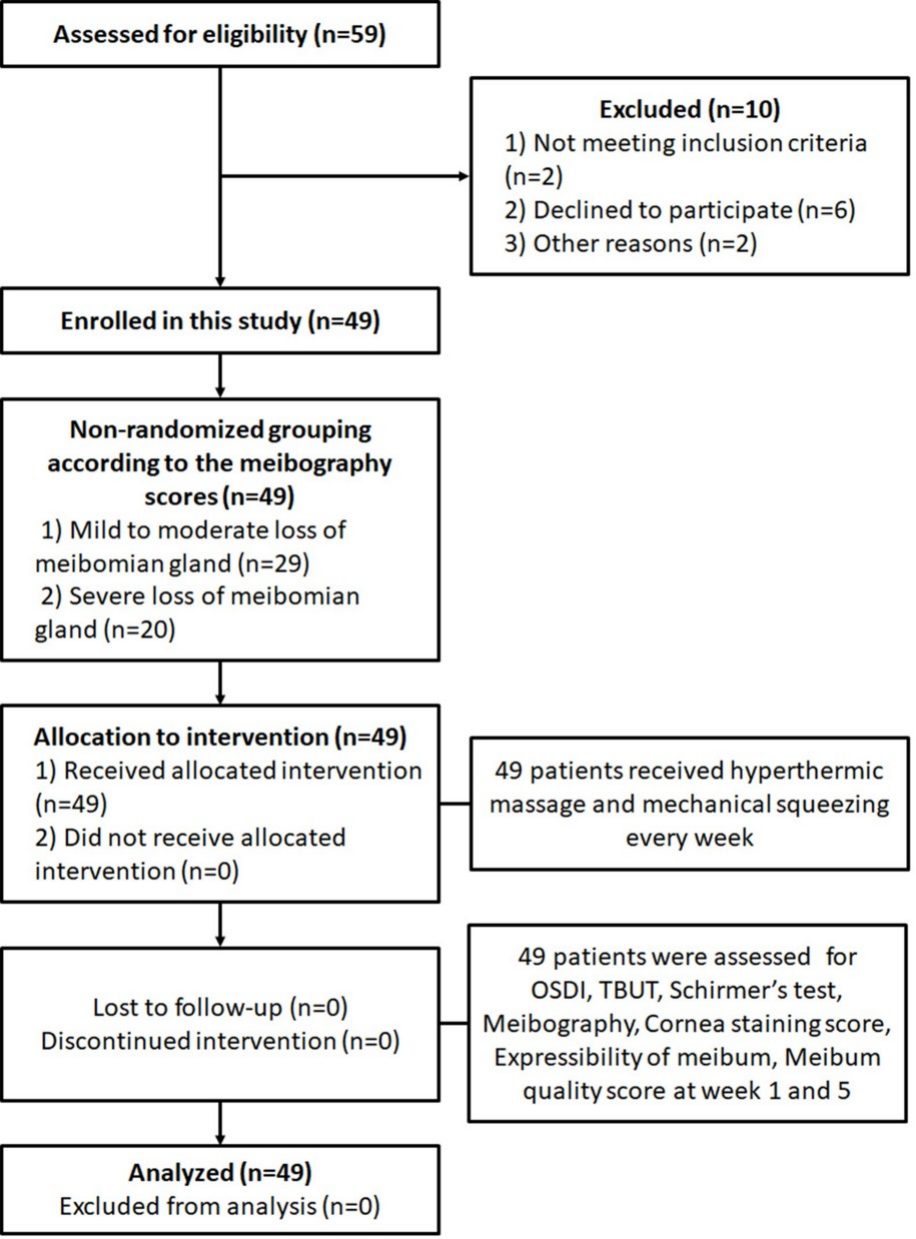
Patient flow of the study.

A total of 59 patients were initially enrolled in the study. Ten patients stopped participating for various reasons: one for epidemic conjunctivitis, one for herpes conjunctivitis, two for the long distance between their residences and the hospital, and six for personal matters. Remaining 49 patients were then categorized into two groups based on meibography scores. Group 1 was with a sum of meibography score of 0 to 4 (mild to moderate dropout of the meibomian gland), having an average age of 50.38 (±11.90) years. Group 2 was with a sum of meibography score of 5 to 6 (severe dropout of the meibomian gland), having an average age of 67.40 (±14.32) years. Both group 1 and 2 were treated for 4 weeks and assessed at the fifth week. Patient flow is indicated in Fig 1. Demographics and other baseline characteristics are shown in Table 1.

**Table 1:** Baseline demographic and clinical characteristics of each group.

	Group 1	Group 2	Total	p-value
Age	50.38 (±11.90)	67.40 (±14.32)	57.33 (±15.34)	***< 0.001^b^
Sex (male:female)	M:12, F:17 (F: 58.6%)	M:9, F:11 (F: 55.0%)	M:21, F:28 (F: 57.1%)	0.801^c^
Meibography score	3.24 (±0.79)	5.15 (±0.37)	4.02 (±1.15)	***< 0.001^b^
OSDI	47.55 (±23.05)	31.95 (±17.94)	41.18 (±22.30)	*0.014^a^
TBUT (seconds)	5.87 (±1.75)	5.88 (±1.32)	5.87 (±1.57)	0.977^a^
Schirmer’s test (mm)	7.26 (±4.34)	6.78 (±4.76)	7.06 (±4.48)	0.521^b^
Expressibility of meibum	22.28 (±11.10)	19.80 (±11.99)	21.27 (±11.42)	0.461^a^
Cornea staining score	1.34 (±0.54)	1.38 (±0.83)	1.36 (±0.66)	0.966^b^
Meibum quality score	2.12 (±0.42)	2.32 (±0.73)	2.20 (±0.57)	0.063^b^

Data are presented as number and mean value±standard deviation. Group 1: mild to moderate dropout of the meibomian gland; Group 2: severe dropout of the meibomian gland.

^a^: Independent samples t-test results.

^b^: Mann-Whitney U test results.

^c^: chi-squared (χ2) test result. OSDI: ocular surface disease index; TBUT: tear film break-up time.

* p<0.05,

*** p<0.001.

After 4 weeks of treatment, the patients in group 1 showed significant changes in meibography score, OSDI, TBUT, expressibility of meibum, and cornea staining score (Table 2). OSDI significantly decreased from 47.55 (±23.05) before treatment to 31.21 (±15.33) after treatment (p<0.001). TBUT, an average of both eyes, significantly increased from 5.87 (±1.75) before treatment to 7.80 (±2.49) after treatment (p<0.001), whereas the expressibility of meibum also increased from 22.28 (±11.10) to 33.33 (±11.84) (p<0.001). Meibography score decreased from 3.24 (±0.79) before treatment to 2.76 (±1.02) after treatment (p<0.01), while cornea staining score also decreased from 1.34 (±0.54) to 1.03 (±0.72) (p<0.05).

**Table 2:** Clinical signs and symptoms before and after hyperthermic and mechanical squeezing treatments in MGD patients with mild to moderate dropout of the meibomian gland (Group 1).

	Before treatment	After treatment	p-value
Meibography score	3.24 (±0.79)	2.76 (±1.02)	**0.005^b^
OSDI	47.55 (±23.05)	31.21 (±15.33)	***< 0.001^a^
TBUT (seconds)	5.87 (±1.75)	7.80 (±2.49)	***< 0.001^b^
Schirmer’s test (mm)	7.26 (±4.34)	7.31 (±3.58)	0.909^b^
Expressibility of meibum	22.28 (±11.10)	33.33 (±11.84)	***< 0.001^a^
Cornea staining score	1.34 (±0.54)	1.03 (±0.72)	*0.039^b^
Meibum quality score	2.12 (±0.42)	2.07 (±0.37)	0.435^b^

Data are presented as number and mean value ±standard deviation.

^a^: Paired t-test results.

^b^: Wilcoxon signed rank test results. OSDI: ocular surface disease index; TBUT: tear film break-up time.

* p<0.05,

** p<0.01,

*** p<0.001.

In case of group 2, the patients showed significant changes in meibography scores, OSDI, TBUT, and expressibility of meibum (Table 3). OSDI in group 2 significantly increased from 31.95 (±17.94) before treatment to 23.65 (±16.22) after treatment (p<0.05), with the increase in TBUT from 5.88 (±1.32) before treatment to 9.03 (±3.04) after treatment (p<0.001) and the expressibility of meibum from 19.80 (±11.99) before treatment to 29.83 (±11.92) after treatment (p<0.001). Meibography score in group 2 as well changed from 5.15 (±0.37) before treatment to 4.75 (±0.64) after treatment (p<0.05).

**Table 3:** Clinical signs and symptoms before and after hyperthermic and mechanical squeezing treatments in MGD patients with severe dropout of the meibomian gland (Group 2).

	Before treatment	After treatment	p-value
Meibography score	5.15 (±0.37)	4.75 (±0.64)	*0.021^b^
OSDI	31.95 (±17.94)	23.65 (±16.22)	*0.049^a^
TBUT (seconds)	5.88 (±1.32)	9.03 (±3.04)	***< 0.001^b^
Schirmer’s test (mm)	6.78 (±4.76)	5.58 (±1.73)	0.226^b^
Expressibility of meibum	19.80 (±11.99)	29.83 (±11.92)	**0.001^a^
Cornea staining score	1.38 (±0.83)	1.15 (±0.56)	0.319^b^
Meibum quality score	2.33 (±0.73)	2.25 (±0.55)	0.492^b^

Data are presented as number and mean value ±standard deviation.

^a^: Paired t-test results.

^b^: Wilcoxon signed rank test results. OSDI: ocular surface disease index; TBUT: tear film break-up time.

* p<0.05,

** p<0.01,

*** p<0.001.

**Fig 2:**
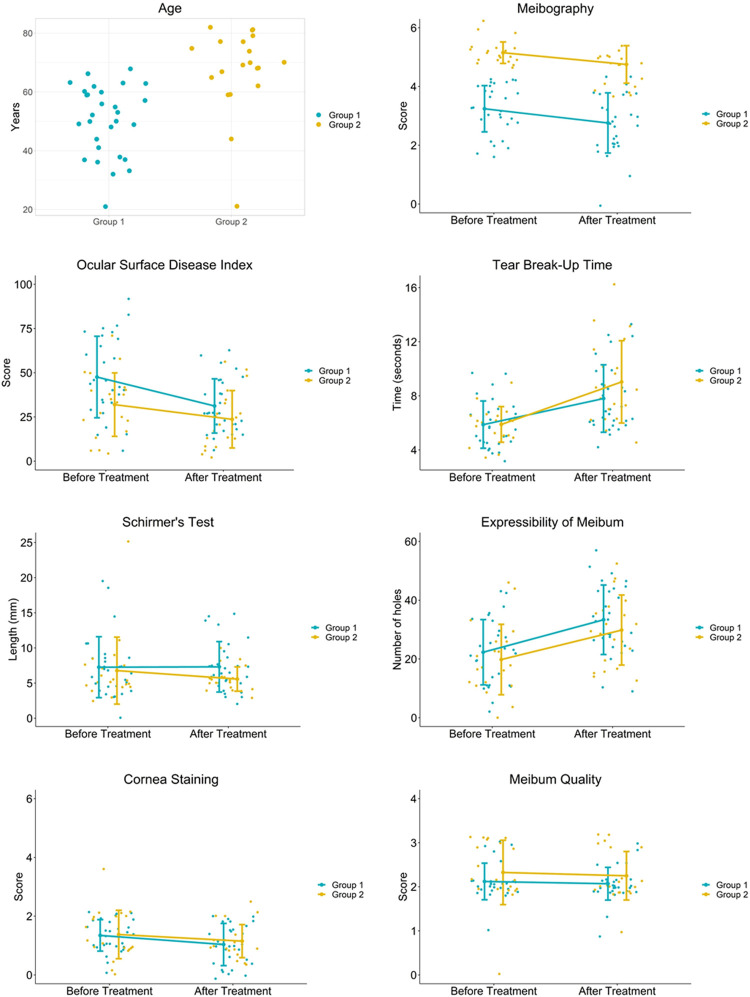
Age and the ocular signs before and after treatments.

The differences of each clinical characteristics before and after treatment were compared to confirm the significance in grouping (Table 4 and Fig 2). The changes in group 1 and group 2 were similar with no significant differences, but there were slight trends observed in each clinical characteristic. Decreases in meibography score, cornea staining score, and meibum quality score were at similar level–meibography score being 0.48 (±0.78) in group 1 and 0.40 (±0.68) in group 2, cornea staining score being 0.31 (±0.85) in group 1 and 0.23 (±0.85) in group2, and meibum quality score being 0.05 (±0.41) in group 1 and 0.08 (±0.61) in group 2. Expressibility of meibum increased at similar level as well in both group 1 and group 2, by 11.12 (±13.58) and 10.03 (±12.02) holes respectively. OSDI decreased in both groups, while group 1 had greater decrease (16.34±17.42) than group 2 (8.30±17.66). TBUT in both groups increased, however group 2 had greater increase (3.15±3.16) than group 1 (1.93±2.46). In case of Schirmer’s test, group 1 increased (0.05±5.45) while group 2 decreased (1.20±3.91).

**Table 4:** Comparison of changes in clinical signs and symptoms between the two groups.

	Group 1	Group 2	p-value
Meibography score	***0.48 (±0.78)	*0.40 (±0.68)	0.864^b^
OSDI	**16.34 (±17.42)	*8.30 (±17.66)	0.121^a^
TBUT (seconds)	***-1.93 (±2.46)	***-3.15 (±3.16)	0.416^b^
Schirmer’s test (mm)	-0.05 (±5.45)	1.20 (±3.91)	0.528^b^
Expressibility of meibum	***-11.12 (±13.58)	**-10.03 (±12.02)	0.773^a^
Cornea staining score	*0.31 (±0.85)	0.23 (±0.85)	0.731^a^
Meibum quality score	0.05 (±0.41)	0.08 (±0.61)	0.450^b^

Data are presented as number and mean value ±standard deviation.

^a^: Independent samples t-test results.

^b^: Mann-Whitney U test results. OSDI: ocular surface disease index; TBUT: tear film break-up time. Asterisks indicate values with significant differences before and after treatment in each group (

* p<0.05,

** p<0.01,

*** p<0.001).

**Fig 3:**
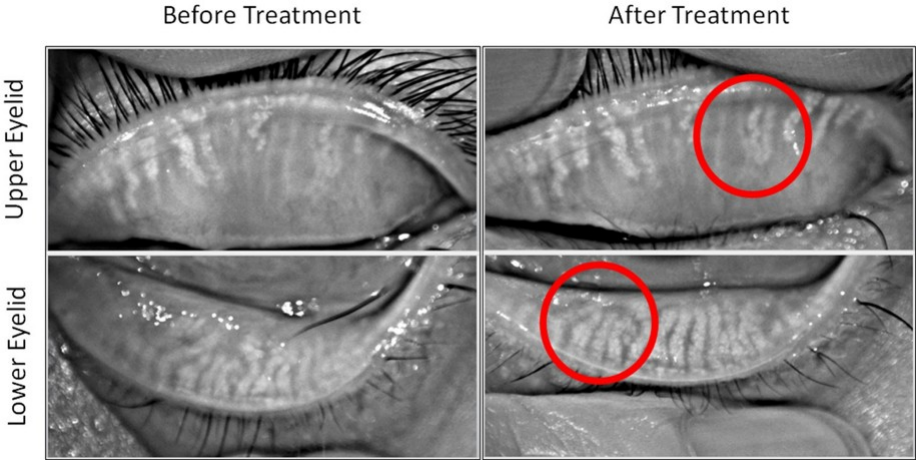
Meibography images of upper and lower eyelids before and after hyperthermic massage and mechanical squeezing.

When the data of all patients in the study were analyzed regardless of the grouping, significant changes were evident in meibography score (p<0.001), OSDI (p<0.001), TBUT (p<0.001), cornea staining score (p<0.05), and expressibility of meibum (p<0.001) (Table 5). Meibography score, OSDI, and cornea staining score showed significant decreases, while TBUT and expressibility of meibum showed significant increase. When the images of ocular conditions were compared before and after treatment, it was well observed that the some of the meibomian glands were regenerated after treatment (Fig 3).

**Table 5:** Clinical signs and symptoms before and after hyperthermic and mechanical squeezing treatment in all participants with MGD.

	Before treatment	After treatment	p-value
Meibography score	4.02 (±1.15)	3.57 (±1.32)	***< 0.001^b^
OSDI	41.18 (±22.30)	28.12 (±15.98)	***< 0.001^a^
TBUT (seconds)	5.87 (±1.57)	8.30 (±2.77)	***< 0.001^b^
Schirmer’s test (mm)	7.06 (±4.48)	6.60 (±3.07)	0.476^b^
Expressibility of meibum	21.27 (±11.42)	31.90 (±11.88)	***< 0.001^a^
Cornea staining score	1.36 (±0.66)	1.08 (±0.66)	*0.022^b^
Meibum quality score	2.20 (±0.57)	2.14 (±0.46)	0.260^b^

Data are presented as number and mean value ±standard deviation.

^a^: Paired t-test results.

^b^: Wilcoxon signed rank test results. OSDI: ocular surface disease index; TBUT: tear film break-up time.

* p<0.05,

*** p<0.001.

## Discussion

Treatment of chronic ocular conditions such as MGD requires well-established strategies which could alleviate symptoms effectively without potential side effects in a long term. Although applying chemical agents may be helpful in controlling symptoms, physical treatments such as hygiene control of eyelids, thermal massage, warm compresses, etc. are usually recommended to the patients with mild to moderate MGD. Numerous studies reported the effectiveness of warm compresses and massage in treating MGD [19, 20], but there is no comparative study with consideration of severity or precise grouping of MGD patients.

This study categorized the patients into two groups according to their meibography scores, and compared the effect of hyperthermic massage and mechanical squeezing in both groups. Although meibography score was the main criterion in grouping, Pearson correlation analysis showed that age was positively correlated with meibomian score and meibum quality score (S2 Table), indicating that older patients would have more severe degree of MGD with greater loss of meibomian gland [21].

The current study observed the improving effects of subjective symptoms and clinical signs by applying hyperthermic massage and mechanical squeezing with no other treatments than artificial tear drops.

When the baselines of group 1 and 2 were compared, there were significant differences in age, OSDI, and meibography score. Since the patients were grouped according to their meibography score, major differences were age and OSDI. The mean age of group 1 was lower than group 2, showing that the severity of meibomian gland loss increased with age. Pearson correlation analysis also showed that age and meibography score have positive correlation with significant difference (p<0.001; S2 Table).

On the other hand, OSDI in group 2 was lower than in group 1, indicating that older patients reported less discomfort in their eyes. Pearson correlation also showed a significant negative correlation between age and OSDI, which suggests that classifying the degree of MGD with OSDI would not be always right and other clinical characteristics should be considered. Based on the previous studies reporting that older people become less sensitive to pain and discomfort [14, 22], mostly due to increased chronic pain [23], diagnosis and treatment on elderly patients require more care.

It was shown that the patients in group 1 (showing mild to moderate dropout of the meibomian gland) had significant decreases in meibography score, OSDI, and cornea staining score with significant increases in TBUT and the expressibility of meibum. On the other hand, the patients in group 2 (showing severe dropout of the meibomian gland) had significant decreases in meibography score and OSDI, with significant increases in TBUT and the expressibility of meibum.

It was hypothesized that such non-pharmaceutical treatment would have minimal effect on the patients with severe MGD, but this study confirmed that hyperthermic massage and mechanical squeezing are still effective in controlling MGD symptoms even with severe loss of meibomian gland.

When the changes in clinical signs before and after treatment were compared between the two groups, no significant differences were observed. The level of changes in meibography score, expressibility of meibum, cornea staining score, and meibum quality score were almost the same in both groups, while OSDI, TBUT, and Schirmer’s test results showed different trends in group 1 and 2.

Decreases in meibography score, cornea staining score, and meibum quality score and increase in expressibility of meibum indicate that hyperthermic massage and mechanical squeezing were effective in controlling MGD, having the same trends in both groups.

In case of OSDI, both groups showed significant decreases after treatment. However, the value in group 2 before treatment was already lower than group 1 and it had declined only about half compared to group 1 after treatment. OSDI after treatment may be as well affected by age-related pain sensitivity changes just as OSDI before treatment [14, 22].

TBUT and Schirmer’s test result are both tear-related characteristics but different trends were observed in the study. TBUT increased in both groups, with greater increase in group 2, while Schirmer’s test results showed an increase in group 1 after treatment and a decrease in group 2 after treatment. Increased TBUT in both groups indicate that application of hyperthermic massage and mechanical squeezing stabilized tear film, and decreased Schirmer’s test in group shows decreased tear volume, with increased tear volume in group 1 after treatment. Pearson’s correlation analysis showed that Schirmer’s test result had significant negative correlations with age and expressibility of meibum (p<0.05; S2 Table), indicating that older patients would have less amount of tear with compensating meibum secretion to maintain tear film. Such compensation seems be related to increased TBUT in both groups [24, 25].

Although group 1 had one more significant clinical sign after treatment than group 2, significant decreases in OSDI and meibography scores, and significant increases in TBUT and the expressibility of meibum were observed for all patients in this study even without grouping. Also, the images of ocular conditions showed that improved eyelid and corneal conditions with meibomian gland regeneration [26].

MGD treatments usually include hygiene control of eyelids, thermal massage, artificial tear drops, topical steroids, and oral antibiotics. Although mechanical squeezing of the meibomian glands is reported to be an effective treatment [27], it is practiced by relatively few clinicians, possibly due to the time required to perform thorough expression of the entire upper and lower eyelids and the associated discomfort [28, 29].

Although there was a study reporting that patients with severe meibomian gland atrophy undergoing treatment with LipiFlow^®^ for 6 months showed no significant improvement in the expressibility of meibum, compared to early MGD patients [30], the current study results showed that there was a significant improvement in the group with severe dropout of the meibomian gland, when hyperthermic massage and mechanical squeezing were applied together. Based on these results, even if the glands are largely lost, it can be expected that the clinical symptoms can be improved if hyperthermic massage and mechanical squeezing treatment is actively performed.

The significance of our study results includes the following: First, classification of MGD patients can be more accurate and precise when using meibography. Currently, MGD patients are commonly being categorized based on OSDI [13], which is subjective and can be altered depending on the patient’s personal sensations. Using meibography in grouping, on the other hand, is objective and based on clear visualization of meibomian glands including dropout states. Second, treating MGD with hyperthermic massage and mechanical squeezing once a week for 4 weeks appears to be effective, even without any other treatment. The current study results showed that hyperthermic massage and mechanical squeezing alleviated symptoms in lowering OSDI, improved quality of meibum in increasing meibum quality score, and regenerated meibomian gland with increased meibography score. Third, we revealed that the treatments used in this study seem to be effective both in patients with mild to moderate and severe loss of meibomian gland, although more effective in mild to moderate MGD group.

Even with the significances, there are still a few limitations in the study. First, the number of patients in group 1 and 2 were different. However, notwithstanding the fact that the number of patients in group 2 was smaller and being older than group 1, such age gap between two groups was not considered to be a selection bias, based on a Korean study in 2015 which revealed a correlation between changes in meibomian gland and age [31]. Second, no further follow-up of the patients was made after the study. There are several studies regarding different follow-up periods after applying warm compresses and massage, and the effect may last for a period from a month [32] to years [33, 34] at maximum, at least not worsening the symptoms [9].

In conclusion, the clinical data collected in this case series suggest the potential efficacy and safety of hyperthermic massage and mechanical squeezing even in the patients with severe loss of meibomian gland as a treatment for meibomian gland dysfunction. In all MGD patients, meibography score, OSDI, and cornea staining score were decreased, while TBUT and expressibility of meibum was increased after treatment. Although it requires time and expertise in performing such treatment, it should be applied to all MGD patients regardless of the severity.

## Supporting information

S1 File Clinical study protocol. (PDF)

S2 File (PDF)

S1 Checklist TREND statement checklist. (DOCX)

S1 Table Individual characteristics and outcomes of patients in group 1 and group 2 before and after treatment. (DOCX)

S2 Table Pearson correlations between clinical characteristics. (DOCX)
