# Supplementary material for: Comparative study on the effect of hyperthermic massage and mechanical squeezing in the patients with mild and severe meibomian gland dysfunction: An interventional case series
Source: PLoS One. 2021 Mar 8;16(3):e0247365. doi: 10.1371/journal.pone.0247365 (PMC7939575; doi:10.1371/journal.pone.0247365)
Supplement: S1 File — (PDF) [file pone.0247365.s001.pdf]

Clinical Study Protocol

Clinical studies to evaluate the therapeutic effect of hyperthermic massage and expression treatments in patients with meibomian gland dysfunction

1. Protocol title

Comparison of the effect of hyperthermic massage and expression treatment according to the classification of patients with meibomian gland dysfunction using meibography

2. Institution name and address of study

Dankook Univ. Hospotal (31116) 201 Manghyang-ro, Dongnam-gu, Cheonan, Choongcheongnamdo, South Korea

3. Purpose and background of the study

Dry eye disease (DED), also known as dry eye syndrome, is one of the most common diagnoses in ophthalmology, and one of the major causes of DED is meibomian gland dysfunction (MGD). MGD is a chronic, diffuse abnormality of the meibomian glands and is commonly characterized by terminal duct obstruction and changes in glandular secretion. The patients with MGD may claim various symptoms depending on the eyelid inflammation and the degree of dry eye. MGD is usually caused by cornification of ductal epithelium and the increased viscosity of meibum. Because the meibomian gland exists under ocular conjunctiva and are only visible under infrared light imaging, as opposed to visible light, it is necessary to perform meibography to diagnose MGD and assess the meibomian gland condition. MGD treatment includes control over eyelid hygiene through patient education, lid scrub, and warm compresses; other treatments include the use of artificial tear drops, topical steroids, oral antibiotics, etc., depending on the patient's symptoms.[1] A previous study indicated that the symptoms in 96% of patients are relieved after 4 weeks of meibomian gland probing. Thermodynamic treatment with Lipiflow® for 3 months also showed an improvement in MGD. In this study, we attempted to assess the effects of hyperthermic massage and expression treatment according to the classification of patients with MGD using meibography.

The present study was designed to investigate the effect of hyperthermic massage and expression treatment according to the classification of patients with meibomian gland dysfunction using meibography.

4. Inclusion and exclusion criteria

Subjects must meet all study inclusion criteria outlined below:

1) Adult males and females age > 18 years with signed informed consent

Subjects meeting any of the following exclusion criteria will not be eligible to participate in the study

1) Patients who have taken systemic medicines such as tetracycline derivatives, anti-histamines, isotretinoin, nutritional supplements, etc.

2) Those who have received steroids within a month

3) Those who have undergone ophthalmic surgery within 3 months

4) Those who have systemic disease with eyelid disorders

A total of 60 MGD patients are target subjects: Group 1) 30 patients with mild MGD; Group 2) 30 patients with sever MGD.

Target subjects are selected according to the following reference, which analyzed the effects of hyperthermic massage on 26 MGD patients (Six-month effects of a thermodynamic treatment for MGD and implications of meibomian gland atrophy. Cornea. 2014 Dec;33(12):1265-70.). The current study aims to compare two groups of patients, hence a total of 60 patients are selected to be target subjects considering

5. Expected Research Period

18 months after IRB approval

6. Research method

- The investigator explains the purpose of this study to patients with meibomian gland dysfunction at Dankook University Hospital.
- Patients are informed to make visits every week for five weeks (a total of five visits), followed by a symptom assessment at each visit.

	Corrected visual acuity	Uncorrected visual acuity	Intraocular pressure	OSDI	TBUT	Schirmer test	Meibography	Ocular surface staining	Expressible meibomian glands and quality
Visit 1 (1 week)	○	○	○	○	○	○	○	○	○
Visit 2 (2 week)		○	○						
Visit 3 (3 week)		○	○						
Visit 4 (4 week)		○	○						
Visit 5 (5 week)	○	○	○	○	○	○	○	○	○

* Evaluation of the study

- Ocular surface disease index score (OSDI)

OSDI is a 12-item scale for the assessment of symptoms related to ocular surface diseases. There is a four-category structure including 3 vision related items, 5 ocular symptom related items, and 4 daily life-related items, with scores graded on a scale of 0-4 (0, none of the time; 1, some of the time; 2, half of the time; 3, most of the time; 4, all the time). OSDI is scored on a scale of 100, with higher scores representing greater disability. OSDI = [(sum of scores for all questions answered) x 100]] / [(total number of questions answered) x 4].

- Have you experienced any of the following during the last week?
  1) Eyes that are sensitive to light?
  2) Eyes that feel gritty? .
  3) Painful or sore eyes?
  4) Blurred vision?
  5) Poor vision?

- Have problems with your eyes limited you in performing any of the following during the last week?
  1) Reading?
  2) Driving at night?
  3) Working with a computer or bank machine (ATM)?
  4) Watching TV?

- Have your eyes felt uncomfortable in any of the following situations during the last week?
  1) Windy conditions?
  2) Places or areas with low humidity (very dry)?
  3) Areas that are air conditioned?

- Patient satisfaction survey (at final visit):

1) Much better
2) Somewhat better
3) No change
4) Somewhat worse
5) Much worse

- Schirmer test

Schirmer test is performed without anesthesia and the tear volume is measured for 5 minutes.

OD (   ) mm / OS (   ) mm

- Tear break up time (TBUT)

TBUT is checked by the elapsed time from a normal blink to the first appearance of a dry spot and is measured 3 times.

OD (   ) sec / OS (   ) sec

- Meibography

Using the noncontact meibography system (Meiboviewer, Visual optics, Choonchun, Korea) the meibomian glands are observed. The dropout of the meibomian glands is graded as 0 (no dropout), 1 (dropout of meibomian glands < 1/3), 2 (1/3 < dropout of meibomian glands <2/3), and 3 (dropout of meibomian glands > 2/3).

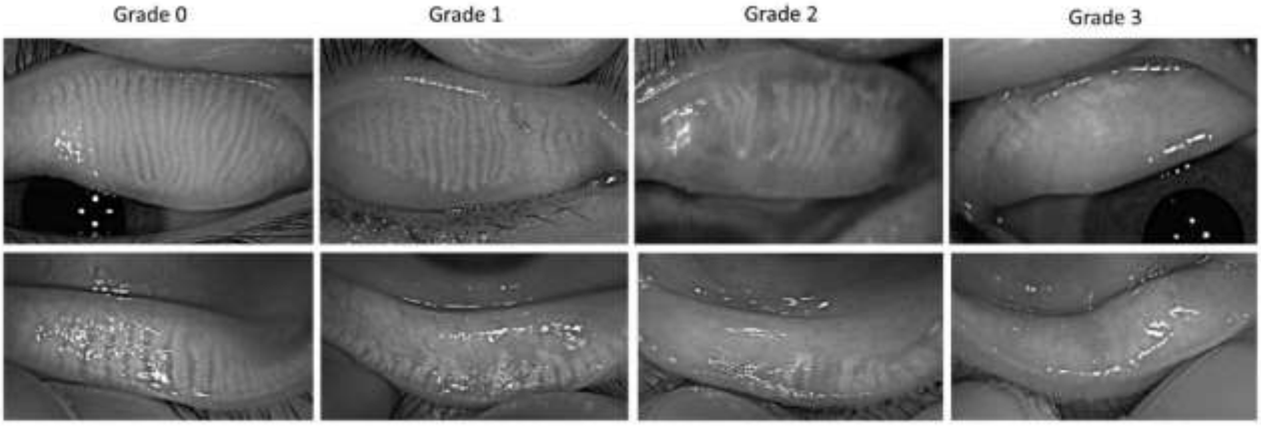

[The dropout of the meibomian glands, 0 (no dropout), 1 (dropout of meibomian glands < 1/3), 2 (1/3 < dropout of meibomian glands <2/3), and 3 (dropout of meibomian glands > 2/3)]

- Ocular surface staining (cornea and conjunctiva)

For Fluorescein corneal and conjunctival staining score scores, 20μl of 1% lissamine green solution is dropped and corneal and conjunctival stainings are evaluated by slit-lamp microscopy. Results are evaluated using Oxford grading scheme from 0 to 6 with increased significance of ocular surface staining.

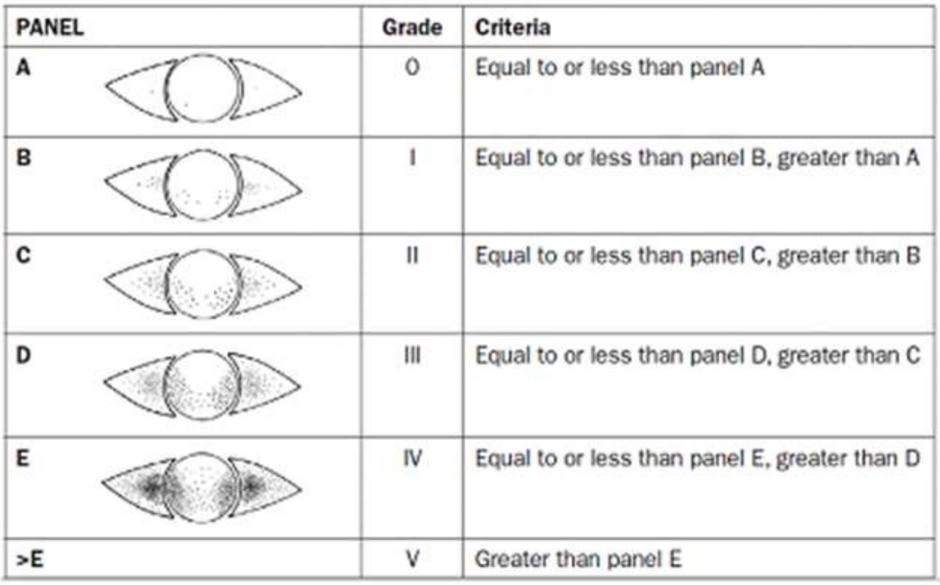

PANEL	Grade	Criteria
A	0	Equal to or less than panel A
B	I	Equal to or less than panel B, greater than A
C	II	Equal to or less than panel C, greater than B
D	III	Equal to or less than panel D, greater than C
E	IV	Equal to or less than panel E, greater than D
>E	V	Greater than panel E

[Oxford grading scheme from 0 to 6 with increased significance of ocular surface staining] [2]

- Expressible meibomian glands and quality (at the first and last visits):

The meibum expressibility is scored as the number which the meibum is induced. The quality of the meibum is graded as 1, clear; 2, cloudy; 3, thick like toothpaste; and 4, no meibum. Lid debris, lid swelling, and lid telangiectasia are scored from 0 (absent) through 4 (very severe) according to the severity of these abnormalities present in each eye.

All patients receive the same treatment as shown below.

1. Preservative-free hyaluronic acid 6 times a day (continue during treatment)
2. Hyperthermic massage for five minutes (Nurieye 5800, Nurieye, Seodong Medical Co., Busan, Korea) and mechanical squeezing (expression) of both upper and lower meibomian glands afterward.

7. Research Schedule

Period	Description
~ 16 months after IRB approval	Patient Screening and Clinical Trial
17 months to 18 months	Analysis and Report Writing

8. Measures to secure research ethics

Patients with dry eye disease should be consulted and consent should be obtained prior to proceeding with the study. After explaining enough information about participation in the study for 30 minutes or more, explain the purpose of the experiment and ask for consent to participate in the experiment.

Subjects who are likely to be socially weak or vulnerable in the process of seeking consent under the Helsinki Declaration are excluded from the test. In the ophthalmic examination case, all personal information except for the sex, age, date of examination, and the first letter of the name is removed to protect the privacy of personal information.

9. References

1. Finis D1, König C, Hayajneh J, Borrelli M, Schrader S, Geerling G. Cornea. 2014 Dec;33(12):1265-70.

2. Babamohamadi H, Nobahar M, Razi J, Ghorbani R. Clin. Nur. Res. 2018; 27(6):714-729.
