# Supplementary material for: Comparative study on the effect of hyperthermic massage and mechanical squeezing in the patients with mild and severe meibomian gland dysfunction: An interventional case series
Source: PLoS One. 2021 Mar 8;16(3):e0247365. doi: 10.1371/journal.pone.0247365 (PMC7939575; doi:10.1371/journal.pone.0247365)
Supplement: S2 File — (PDF) [file pone.0247365.s002.pdf]

연 구 계 획 서

연구과제명	국 문	마이봄샘촬영술을 이용한 마이봄샘 기능장애 환자 분류에 따른 온열 및 짜줌 치료효과 비교
	영 문	Comparison of the effect of hyperthermic massage and expression treatment according to the classification of patients with meibomian gland dysfunction using meibography

1. 연구의 명칭

마이봄샘 기능장애 환자를 마이봄샘촬영술을 이용하여 마이봄샘 소실의 심한 정도에 따라 두 군으로 분류 후 눈꺼풀 온열 및 마이봄샘 짜줌 치료 효과가 군에 따라 차이가 있는 지 알아보고한 한다.

2. 연구의 실시기관명 및 주소

단국대학교병원, 충남 천안시 동남구 망향로 201

3. 연구 책임자, 담당자, 공동연구자 성명 및 직명

		소 속	직 위	성 명	전공분야	e-mail
연 구 자	책임연구자	안과	조교수	조경진	각막, 백내장	perfectcure@hanmail.net
	공동연구자					
	연구담당자	안과	연구간호사	신선필	각막, 백내장	ssp810109@hanmail.net
	코디네이터	안과	연구원	이효순	각막, 백내장	hyosun33@hanmail.net

4. 연구비 지원기관명 및 주소

단국대학교병원 레이저중개임상시험지원센터, 충청남도 천안시 동남구 망향로 201

5. 연구의 목적 및 배경

안구건조증은 가장 흔한 안과 질환이며 이러한 안구건조증의 가장 중요한 원인 중 하나가 마이봄샘 기능장애 (Meibomian gland dysfunction) 이다.

- 마이봄샘은 위, 아래 눈꺼풀에 존재하는 피지샘으로 안구표면에 기름성분을 분비함으로써 눈물막의 지질층을 형성하고 이 지질층은 눈물의 증발을 방지하는 역할을 한다.
- 안구건조증의 경우 크게는 눈물분비부족이나 눈물증발과다로 인한 두 가지 종류로 나뉘며, 특히 눈물증발과다로 인한 증발성 안구건조증의 경우, 단순하게 눈물만 부족한 경우는 14.5%에 그치며 마이봄샘 지질 분비가 부족한 마이봄샘 기능장애로 인한 경우가 많다.[1]

- 마이봄샘은 눈꺼풀 결막밑에 존재하기 때문에 가시광에서는 보이지 않으며 적외선 이미징을 통해서만 보이기 때문에 마이봄샘 기능장애를 진단하기 위해서는 마이봄샘촬영술(Meibography)가 필요하다.[2]

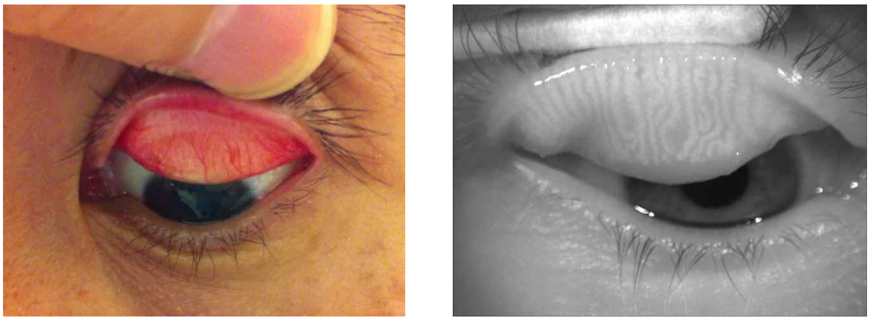

[마이봄샘은 가시광선(좌)에서는 보이지 않으며 적외선(우)에는 관찰됨]

- 본 연구의 목적은 안구건조증의 주요 원인인 마이봄샘기능장애 환자에서 마이봄샘촬영술을 사용하여 마이봄샘의 상태를 평가하고 이에 따라 마이봄샘 기능장애의 심한 정도에 따라 두 군으로 나누었을 때 마이봄샘 기능저하의 통상적인 치료법인 온열 및 짜줌의 효과[3]가 군에 따라 차이를 보이는지를 확인하여 마이봄샘촬영술의 유용성을 평가하고자 한다.

6. 연구 대상

대상자의 선정기준 : 단국대학교병원 안과를 내원한 18세 이상의 마이봄샘 기능장애 환자를 대상으로 한다.

제외기준 : 최근 3개월 이내 전신복용약 사용 (tetracycline derivatives, antihistamines, isotretinoin, nutritional supplements), 최근 1달 이내 steroid를 사용하는 경우, 최근 3달 이내 안과적 수술을 시행받은 경우, 눈꺼풀의 이상을 유발하는 전신 질환이 있는 경우로 한다.

목표대상자 : 마이봄샘 기능장애 환자 60명을 목표 대상자로 한다.
(1군 – 마이봄샘의 소실이 심하지 않은 환자 30명,
2군 – 마이봄샘의 소실이 심한 환자 30명)

* 목표 대상자 산출 근거 – 아래의 논문에서 마이봄샘 기능장애 환자 26명의 온열치료효과를 분석하였다. 본 연구에서는 마이봄샘 기능장애 환자를 두 군으로 나누어야하고, 피험자 탈락 등을 고려하여 60명을 목표 대상자로 한다.

참고 논문 : Six-month effects of a thermodynamic treatment for MGD and implications of meibomian gland atrophy. Cornea. 2014 Dec;33(12):1265-70.

7. 예상연구기간

IRB 승인 후 18개월

8. 연구방법 - 임상시험에 제시된 필수항목을 자유롭게 기술

- 단국대학교병원 안과에 안구건조증 때문에 내원한 환자 중 마이봄샘 기능장애 환자를 대상으로 본 연구의 목적을 설명하고 동의서를 받아 임상시험을 시작한다.
- 환자는 1주일 간격으로 총 5회 방문하게 되고 방문시마다 다음의 검사를 시행받는다.

	교정 시력	나안 시력	안압	OSDI	TBUT	셔머 test	meibo-graphy	Ocular surface staining(각막, 결막)	expressible meibomian glands and quality
1회방문 (1주)	o	o	o	o	o	o	o	o	o
2회방문 (2주)		o	o						
3회방문 (3주)		o	o						
4회방문 (4주)		o	o						
5회방문 (5주)	o	o	o	o	o	o	o	o	o

* 안구표면질환지수 (Ocular surface disease index score, OSDI)
안구표면질환지수는 안구표면질환에 대한 평가이다. 총 12문항이며, 시력관련 항목이 3가지, 안구증상관련 항목이 5가지, 일상생활지장과 관련된 항목이 4가지로 구성되어있다. 증상이 없으면 0점, 가끔 증상이 있으면 1점, 반나절정도 증상이 있으면 2점, 대부분 증상이 있으면 3점, 하루종일 증상이 있으면 4점으로 각 5점 척도로 평가한다.
안구표면 질환지수의 점수는 다음과 같이 계산되어 0점에서 100점 까지이며 점수가 높을수록 증상이 심한 상황이다.
OSDI 점수는= [대답한 모든 항목의 합계×100)] / [대답한 질문의 총 수×4]

다음의 상황에 대해서 답변해 주시기 바랍니다.
(상황과 관련없는 문항은 답변하지 않으셔도 됩니다.)

(0) 전혀없음 (1) 가끔 (2) 반정도 (3) 대부분 (4) 항상

지난 일주일간 다음의 증상은 얼마나 자주 느끼셨나요?

1) 불빛을 보면 눈이 부신다. (0) (1) (2) (3) (4)
2) 모래알이 굴러다니는 느낌이 든다. (0) (1) (2) (3) (4)
3) 눈이 아프거나 따갑다. (0) (1) (2) (3) (4)
4) 시야가 흐리다. (0) (1) (2) (3) (4)
5) 시력이 떨어져 잘 보이지 않는다. (0) (1) (2) (3) (4)
6) 눈이 가렵고 간지럽다. (0) (1) (2) (3) (4)
7) 아침에 충혈이 심하다. (0) (1) (2) (3) (4)
8) 아침에 눈이 끈적거려 잘 안떠진다. (0) (1) (2) (3) (4)

지난 일주일간 눈 때문에 다음의 일상생활에 얼마나 지장이 있으셨나요?

9) 독서 (0) (1) (2) (3) (4)
10) 야간 운전 (0) (1) (2) (3) (4)
11) 컴퓨터 은행 출금작업 (0) (1) (2) (3) (4)
12) 텔레비전 (0) (1) (2) (3) (4)

* 환자 만족도 평가 (마지막 방문 시 평가)
1. 많이 좋아졌다
2. 조금 좋아졌다
3. 변화 없다
4. 조금 나빠졌다
5. 많이 나빠졌다.

* 셔머 test
마취 없는 상태에서 셔머용지를 이용하여 5분간 측정한다.
(Tear secretion - Schirmer test (without anesthesia) for 5 minute)
OD ( ) mm / OS ( )

* 눈물막 파괴시간 측정 (Tear break up time)
황색 필터를 이용한 코발트블루 광원의 세극등 조명하에 플루오레세인 염색후 눈을 깜박이게 한 후 마지막으로 깜박인 시점부터 플루오레세인으로 염색된 눈물층에서 검은점, 줄의형태 또는 플루오레세인의 결손이 관찰될 때 까지의 시간을 초 단위로 측정한다. 측정결과는 3회반복 측정하여 평균값을 사용한다.
TBUT (fluoresceine dye) OD ( ) sec / OS ( ) sec

* 마이봄샘 촬영술 (Meibography)
마이봄샘 촬영장비(마이보뷰어, ㈜비주얼옵틱스, 대한민국 강원도 춘천)를 사용하여 마이봄샘을 촬영하여 아래의 그림과 같이 마이봄샘의 소실(Dropout) 비유에 따라 grade 0 ~ grade 3 까지 4단계로 분유한다.

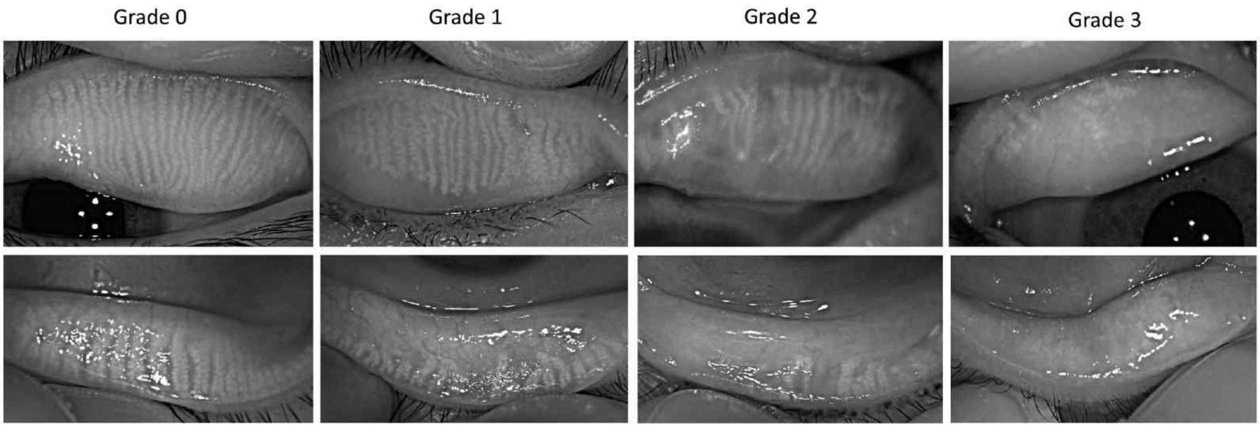

[Grade 0: 마이봄샘의 소실이 없음, Grade 1: 마이봄샘의 소실이 1/3 이하, Grade 2:마이봄샘의 소실이 1/3이상 ~ 2/3이하, 마이봄샘 소실이 2/3 이상]

* 안구표면 염색 검사(ocular surface staining, 각막, 결막)
  - 각막염색검사 : 황색필터를 이용한 코발트블루광원의 세극등조명하에 플루오레세인 염색 후 각막염색정도를 아래의 oxford grading system에 따라 6점 척도로 평가한다.[4]
  - 결막염색검사 : 세극등하에서 리사민그린염색 후 결막을 비측과 이측 영역으로 나누어 비측결막 및 이측결막염색정도를 oxford grading system에 따라 6점 척도로 평가한다.[4] 이측결막평가를 위하여 시험대상자는 코방향을 보도록 하며, 비측결막평가를 위하여 귀방향을 보도록한다.

* Expressible meibomian glands and quality (처음과 마지막 방문시 평가)
  - Expressible MGD의 개수: 위, 아래 각각 짰을 때 나오는 구멍의 수.
  - Meibum의 quality
    1. clear 2. cloudy 3. toothpaste 4. no meibum

- 모든 환자는 아래와 같이 동일한 치료를 받는다.
  1. 히알루론산 성분의 무방부제 인공누액 하루 6회 점안 (치료기간 동안 계속)
  2. 매 방문시마다 온열찜질 (5분간, 누리아이 5800사용 (Nuri Eye, 서동메디칼, 대한민국 부산 진구) 후 면봉을 이용하여 위, 아래눈꺼풀의 마이봄샘을 짜줌 (Expression).

- 마이봄샘 기능장애 환자를 처음 방문시 측정한 위눈꺼풀과 아래눈꺼풀 마이봄점수(Meiboscore)의 합이 0 ~ 4인군 (마이봄샘 소실이 적은 군) 과, 5 ~ 6 인 군 (마이봄샘 소실이 심한 군)으로 나누어 두 군간의 OSDI, 만족도, 셔머Test, TBUT, Meiboscore, 안구표면염색검사, Expressible meibomian glands and quality를 치료 전과 치료 후에 비교 분석한다. Meiboscore가 심한 눈을 기준으로 한다.
  * 분석 방법은 paired t-test를 사용한다.

9. 연구수행일정표

기 간	내 용
IRB 승인 후 ~ 16 개월	대상환자 선별 검체 수집
17 개월 ~ 18개월	분석 및 각 군 비교

10. 연구의 윤리성 확보를 위한 방안 (헬싱키선언 준수는 반드시 명시되어야 하며, 그 외에도 해당 연구의 윤리성 확보를 위해 필요한 사항을 기술할 것)

안구건조증 환자중 마이봄샘 기능장애 환자를 대상으로 하며, 검사를 진행하기 전 임상시험 대한 동의를 구한다. 연구참여에 대한 충분한 정보를 30분이상 자세히 설명하여 실험의 목적을 설명한 후에 실험에 참여하겠다는 동의를 구한다.
헬싱키선언에 따라 동의를 구하는 과정에서 사회적 약자나 취약자가 될 가능성이 있는 피험자는 시험에서 배제한다. 안과검사 증례지에서 환자의 성별, 나이, 검사날짜, 이름의 첫글자 이외의 모든 개인정보는 제거하여 개인정보 비밀을 보호한다.

11. 참고 문헌

1) Finis D1, König C, Hayajneh J, Borrelli M, Schrader S, Geerling G. Cornea. 2014 Dec;33(12):1265-70.
2) Lemp MA, Crews LA, Bron AJ, Foulks GN, Sullivan BD. 2012 May;31(5):472-8.
3) Ding J, Sullivan DA. 2012 Apr;47(7):483-90.
4) Babamohamadi H, Nobahar M, Razi J, Ghorbani R. Clin. Nur. Res. 2018; 27(6):714-729. (Oxford grading scheme)

12. 피험자 설명문 및 동의서 - 필요시
파일로 첨부함

13. 피험자동의면제사유서 - 필요시
해당사항 없음

14. 증례기록서
파일로 첨부함

15. 피험자 모집 방법과 문건 - 필요시
해당사항 없음

16. 피험자 보상규약 - 필요시
해당사항 없음
