# Supplementary material for: Comparative study on the effect of hyperthermic massage and mechanical squeezing in the patients with mild and severe meibomian gland dysfunction: An interventional case series
Source: PLoS One. 2021 Mar 8;16(3):e0247365. doi: 10.1371/journal.pone.0247365 (PMC7939575; doi:10.1371/journal.pone.0247365)
Supplement: S1 Table — (DOCX) [file pone.0247365.s004.docx]

S1 Table. Individual characteristics and outcomes of patients in Group 1 and Group 2 before and after treatment

| Group 1 | | | Before Treatment | | | | | | | After Treatment | | | | | | |
| --- | --- | --- | --- | --- | --- | --- | --- | --- | --- | --- | --- | --- | --- | --- | --- | --- |
| # | Sex | Age | MS | OSDI | TBUT | ST | EM | CS | MQ | MS | OSDI | TBUT | ST | EM | CS | MQ |
| m01 | F | 50 | 4 | 40 | 4.615 | 5 | 10.5 | 1.5 | 2 | 2 | 33 | 6.65 | 3.5 | 43 | 1 | 2 |
| m05 | M | 53 | 3 | 65 | 4.515 | 19.5 | 11 | 1 | 1 | 4 | 15 | 7.2 | 2 | 22 | 1 | 2.5 |
| m06 | M | 60 | 2 | 27 | 4.085 | 8 | 11 | 1 | 2 | 2 | 13 | 6.4 | 13.5 | 10.5 | 2 | 1.5 |
| m07 | M | 37 | 4 | 19 | 8.835 | 18.5 | 5 | 1 | 2 | 4 | 15 | 11.12 | 6 | 33 | 0 | 1 |
| m09 | F | 44 | 4 | 35 | 6.8 | 7.5 | 10.5 | 1.5 | 2 | 4 | 27 | 8.85 | 7.5 | 46.5 | 0 | 2 |
| m10 | F | 60 | 2 | 46 | 3.745 | 7 | 14 | 2 | 3 | 2 | 17 | 5.795 | 10.5 | 24 | 1.5 | 2.5 |
| m13 | M | 21 | 4 | 77 | 5 | 7.5 | 38 | 1 | 2 | 2 | 46 | 5.13 | 3 | 27.5 | 1.5 | 2 |
| m14 | F | 49 | 2 | 42 | 5.025 | 3.5 | 42.5 | 1 | 2 | 3 | 27 | 4.2 | 6 | 43 | 1 | 2 |
| m16 | F | 36 | 2 | 44 | 5.025 | 6 | 19.5 | 2 | 2 | 2 | 23 | 6.17 | 6.5 | 44 | 1 | 2 |
| m18 | F | 57 | 4 | 56 | 6.24 | 3.5 | 25.5 | 0.5 | 2 | 4 | 63 | 5.52 | 15 | 41 | 1 | 2 |
| m19 | F | 63 | 4 | 69 | 3.165 | 0 | 23.5 | 1 | 2 | 4 | 52 | 5.82 | 3.5 | 44.5 | 1.5 | 2 |
| m20 | M | 33 | 3 | 73 | 4.455 | 14.5 | 15 | 1 | 2 | 3 | 38 | 11.995 | 5.5 | 20 | 1.5 | 2 |
| m22 | F | 32 | 3 | 40 | 3.795 | 3 | 29.5 | 2 | 2.5 | 2 | 27 | 6.145 | 14.5 | 34.5 | 2 | 2.5 |
| m25 | F | 55 | 4 | 29 | 8.165 | 8.5 | 19.5 | 1 | 2 | 4 | 23 | 13.295 | 6 | 29 | 0.5 | 2 |
| m26 | F | 63 | 4 | 92 | 6.665 | 6.5 | 22 | 1.5 | 2 | 4 | 60 | 6.075 | 14 | 51.5 | 1.5 | 2 |
| m28 | M | 59 | 4 | 71 | 3.98 | 7 | 35 | 1.5 | 2 | 3 | 48 | 12.42 | 7.5 | 23 | 2 | 3 |
| m31 | F | 66 | 3 | 60 | 9.685 | 10.5 | 33.5 | 2 | 2 | 2 | 38 | 5.49 | 7 | 35.5 | 0.5 | 2 |
| m32 | F | 38 | 3 | 38 | 9.64 | 7.5 | 27.5 | 1 | 2 | 2 | 27 | 12.5 | 9.5 | 28.5 | 0 | 2 |
| m36 | M | 48 | 2 | 58 | 5.865 | 4.5 | 43 | 1.5 | 2 | 2 | 38 | 5.86 | 7.5 | 26.5 | 0.5 | 2 |
| m38 | F | 63 | 3 | 73 | 6.595 | 10.5 | 21 | 1.5 | 2 | 3 | 46 | 8.515 | 4 | 33.5 | 2 | 2 |
| m41 | F | 68 | 4 | 15 | 7.195 | 8.5 | 23 | 2 | 2 | 4 | 21 | 8.61 | 7.5 | 26 | 1.5 | 2 |
| m42 | M | 37 | 4 | 75 | 5.715 | 8.5 | 0 | 0 | 2 | 3 | 56 | 7.005 | 5.5 | 28.5 | 2 | 2 |
| m45 | F | 62 | 3 | 6 | 6.165 | 5.5 | 11 | 1.5 | 3 | 3 | 6 | 8.02 | 4.5 | 27 | 0 | 2 |
| m46 | F | 41 | 2 | 19 | 7.77 | 4.5 | 33 | 1.5 | 2 | 0 | 27 | 9.84 | 7.5 | 57 | 1 | 2 |
| m47 | M | 56 | 3 | 33 | 7.03 | 3 | 28 | 2 | 3 | 3 | 18 | 6.145 | 3 | 46.5 | 0.5 | 2 |
| m50 | F | 52 | 3 | 83 | 5.505 | 4 | 22 | 2 | 2 | 3 | 21 | 6.86 | 6.5 | 38 | 1 | 2 |
| m51 | M | 50 | 4 | 33 | 5.625 | 9 | 35.5 | 0.5 | 2 | 2 | 44 | 9.89 | 8.5 | 49 | 0 | 2 |
| m54 | M | 59 | 4 | 15 | 4.565 | 4 | 10 | 2 | 3 | 3 | 15 | 6.295 | 11.5 | 9 | 2 | 3 |
| m56 | M | 49 | 3 | 46 | 4.69 | 5 | 24.5 | 1 | 2 | 1 | 21 | 8.335 | 5 | 24.5 | 0 | 2 |

| Group 2 | | | | | Before Treatment | | | | | | | After Treatment | | | | | | |
| --- | --- | --- | --- | --- | --- | --- | --- | --- | --- | --- | --- | --- | --- | --- | --- | --- | --- | --- |
| # | | Sex | Age | | MS | OSDI | TBUT | ST | EM | CS | MQ | MS | OSDI | TBUT | ST | EM | CS | MQ |
| m03 | 11033930 | | | F | 21 | 5 | 40 | 6.145 | 25 | 3.5 | 2 | 5 | 8 | 6.28 | 10 | 19.5 | 2 | 2 |
| m08 | 10911347 | | | M | 70 | 5 | 40 | 6.15 | 2.5 | 16.5 | 1 | 5 | 40 | 6.305 | 4 | 52.5 | 0.5 | 1 |
| m11 | 07897027 | | | M | 79 | 5 | 27 | 6.635 | 7 | 8.5 | 3.5 | 6 | 25 | 8.77 | 8 | 15.5 | 1 | 3 |
| m12 | 04307152 | | | M | 65 | 5 | 35 | 6.52 | 5.5 | 0 | 0 | 4 | 21 | 7.02 | 6 | 38 | 1 | 2 |
| m15 | 01072990 | | | M | 67 | 5 | 23 | 4.135 | 7.5 | 12 | 1.5 | 5 | 27 | 5.43 | 6 | 32 | 1 | 3 |
| m17 | 01780626 | | | M | 82 | 5 | 4 | 5.235 | 5 | 22.5 | 0 | 5 | 17 | 10.15 | 5 | 36.5 | 1 | 2 |
| m21 | 08990549 | | | M | 81 | 5 | 6 | 3.425 | 6 | 12 | 1 | 4 | 2 | 7.8 | 5 | 28.5 | 1 | 3 |
| m24 | 03718759 | | | F | 68 | 5 | 33 | 5.06 | 11 | 16 | 2 | 4 | 27 | 13.145 | 7.5 | 23.5 | 2.5 | 2 |
| m27 | 05392571 | | | F | 44 | 5 | 25 | 5.075 | 7.5 | 46 | 2 | 5 | 33 | 16.245 | 5.5 | 46.5 | 1 | 2 |
| m29 | 09401080 | | | F | 74 | 5 | 50 | 6.525 | 4.5 | 33 | 1 | 5 | 35 | 9.65 | 4.5 | 47.5 | 1.5 | 2 |
| m30 | 05084111 | | | F | 68 | 5 | 58 | 6.825 | 4.5 | 26 | 1 | 4 | 13 | 12.21 | 8.5 | 35 | 0.5 | 2 |
| m33 | 11195232 | | | F | 81 | 5 | 42 | 8.98 | 5.5 | 11 | 1 | 4 | 48 | 8.46 | 4 | 12.5 | 2 | 2 |
| m34 | 11287427 | | | F | 69 | 5 | 38 | 6.2 | 5 | 18 | 1 | 5 | 56 | 7.395 | 5 | 26.5 | 1 | 2 |
| m37 | 08881436 | | | F | 77 | 5 | 50 | 5.755 | 4 | 11 | 2 | 5 | 6 | 11.43 | 5 | 14 | 1 | 3 |
| m39 | 04085856 | | | M | 70 | 5 | 38 | 6.18 | 3 | 26 | 0.5 | 5 | 13 | 10.05 | 5.5 | 16.5 | 0.5 | 2 |
| m43 | 06737773 | | | F | 75 | 6 | 13 | 6.03 | 5 | 17 | 2 | 6 | 8 | 13.565 | 5.5 | 15 | 1 | 3 |
| m49 | 00804606 | | | F | 77 | 6 | 6 | 7.81 | 8.5 | 21 | 2 | 5 | 13 | 8.62 | 5.5 | 35.5 | 2 | 2 |
| m53 | 00780849 | | | F | 62 | 5 | 23 | 6.375 | 4.5 | 44 | 1 | 4 | 25 | 7.295 | 4 | 40 | 0.5 | 2 |
| m55 | 10639234 | | | M | 59 | 6 | 17 | 4.91 | 5 | 29.5 | 1 | 4 | 4 | 6.22 | 4 | 29.5 | 1 | 2 |
| m57 | 04747457 | | | M | 59 | 5 | 71 | 3.65 | 9 | 22.5 | 2 | 5 | 52 | 4.545 | 3 | 32 | 1 | 3 |

MS: Meibography score, OSDI: Ocular surface disease index, TBUT: Tear break-up time, ST: Schirmer’s test, EM: Expressibility of meibum, CS: Cornea staining score, MQ: Meibum quality score.
