# Supplementary material for: Comparative study on the effect of hyperthermic massage and mechanical squeezing in the patients with mild and severe meibomian gland dysfunction: An interventional case series
Source: PLoS One. 2021 Mar 8;16(3):e0247365. doi: 10.1371/journal.pone.0247365 (PMC7939575; doi:10.1371/journal.pone.0247365)
Supplement: S2 Table — (DOCX) [file pone.0247365.s005.docx]

S2 Table. Pearson correlations between clinical characteristics

|  | Age | Meibography score | OSDI | TBUT | Schirmer’s test | Cornea staining score | Expressibility of meibum | Meibum quality score |
| --- | --- | --- | --- | --- | --- | --- | --- | --- |
| Age | 1 | ***0.486 | *-0.297 | 0.072 | **-0.388 | 0.042 | -0.128 | *0.293 |
|  |  | (0.000) | (0.038) | (0.624) | (0.006) | (0.776) | (0.381) | (0.041) |
| Meibography score | ***0.486 | 1 | *-0.290 | 0.062 | -0.012 | -0.051 | -0.170 | 0.138 |
|  | (0.000) |  | (0.043) | (0.670) | (0.932) | (0.727) | (0.243) | (0.345) |
| OSDI | *-0.297 | *-0.290 | 1 | -0.143 | 0.072 | -0.068 | 0.123 | -0.261 |
|  | (0.038) | (0.043) |  | (0.328) | (0.624) | (0.642) | (0.398) | (0.070) |
| TBUT | 0.072 | 0.062 | -0.143 | 1 | 0.140 | -0.006 | 0.016 | -0.124 |
|  | (0.624) | (0.670) | (0.328) |  | (0.338) | (0.965) | (0.915) | (0.396) |
| Schirmer’s test | **-0.388 | -0.012 | 0.072 | 0.140 | 1 | 0.087 | *-0.348 | -0.171 |
|  | (0.006) | (0.932) | (0.624) | (0.338) |  | (0.550) | (0.014) | (0.240) |
| Cornea staining score | 0.042 | -0.051 | -0.068 | -0.006 | 0.087 | 1 | -0.009 | ***0.551 |
|  | (0.776) | (0.727) | (0.642) | (0.965) | (0.550) |  | (0.949) | (0.000) |
| Expressibility of meibum | -0.128 | -0.170 | 0.123 | 0.016 | *-0.348 | -0.009 | 1 | -0.082 |
|  | (0.381) | (0.243) | (0.398) | (0.915) | (0.014) | (0.949) |  | (0.577) |
| Meibum quality score | *0.293 | 0.138 | -0.261 | -0.124 | -0.171 | ***0.551 | -0.082 | 1 |
|  | (0.041) | (0.345) | (0.070) | (0.396) | (0.240) | (0.000) | (0.577) |  |

Significances are displayed in parenthesis. OSDI: ocular surface disease index; TBUT: tear film break-up time. * p<0.05, ** p<0.01, *** p<0.001.
